# SRAdb: query and use public next-generation sequencing data from within R

**DOI:** 10.1186/1471-2105-14-19

**Published:** 2013-01-17

**Authors:** Yuelin Zhu, Robert M Stephens, Paul S Meltzer, Sean R Davis

**Affiliations:** 1 Genetics Branch, National Cancer Institute, National Institutes of HealthBethesda, MD 20892, USA; 2 Advanced Biomedical Computing Center, National Cancer Institute-Frederick, SAIC-Frederick Inc., Frederick, MD 21702, USA

## Abstract

**Background:**

The Sequence Read Archive (SRA) is the largest public repository of sequencing data from the next generation of sequencing platforms including Illumina (Genome Analyzer, HiSeq, MiSeq, .etc), Roche 454 GS System, Applied Biosystems SOLiD System, Helicos Heliscope, PacBio RS, and others.

**Results:**

SRAdb is an attempt to make queries of the metadata associated with SRA submission, study, sample, experiment and run more robust and precise, and make access to sequencing data in the SRA easier. We have parsed all the SRA metadata into a SQLite database that is routinely updated and can be easily distributed. The SRAdb R/Bioconductor package then utilizes this SQLite database for querying and accessing metadata. Full text search functionality makes querying metadata very flexible and powerful. Fastq files associated with query results can be downloaded easily for local analysis. The package also includes an interface from R to a popular genome browser, the Integrated Genomics Viewer.

**Conclusions:**

SRAdb Bioconductor package provides a convenient and integrated framework to query and access SRA metadata quickly and powerfully from within R.

## Background

High throughput next-generation sequencing (NGS) technologies are very rapidly becoming standard tools in biology. The data generated are large and extremely rich. As such, the Sequence Read Archive (SRA, [1]) has been set up at NCBI in the United States, EMBL in Europe, and DDBJ in Japan to capture these data in public repositories in much the same spirit as MIAME-compliant microarray databases like NCBI Gene Expression Omnibus (GEO) and EBI ArrayExpress. As these public data resources continue to grow, the opportunities to leverage them for comparison to private data or to generate novel hypotheses will also grow.

Searching for and retrieving data from the SRA database is becoming a common task for those involved in all aspects of biological research. The SRA website offers interactive search functionality that is often adequate to find datasets of interest. SRAdb is not meant to replace these workflows, but to augment them with additional search capabilities and programmatic access to SRA data and metadata. The package has been developed to provide a convenient and integrated framework to query and access SRA metadata quickly and powerfully from within R [2] and Bioconductor [3]. In addition, SRAdb features functionality to determine availability of sequence files and to download files of interest in bulk based on the results of queries on the metadata. The data consumer can then use R, Bioconductor, or third- party tools to proceed with analysis. Finally, because of our own need to visualize processed data in bulk, particularly in the context of experimental metadata, the package provides functions to let R interacts with a powerful and feature-rich genome browser - the Integrative Genomics Viewer (IGV, [4]) for data visualization and exploration.

SRA does not currently store aligned reads or any other processed data that might rely on alignment to a reference genome. However, the NCBI GEO database does often contain aligned reads for sequencing experiments and the SRAdb package can be used to facilitate access to these data as well. Since SRA is a continuously growing repository, the SRAdb SQLite file is updated regularly.

## Design

Submission of SRA data includes experimental data (typically sequence data files) and metadata describing the experiments and biologicals. The metadata are composed of five main data objects: submission, study, sample, experiment and run. Each object is defined by an XML schema and an XML submission contains one or more metadata objects that are related to each other. After submission, an unique accession number is issued for each object. The SRAdb package maps each of the five main data objects to a set of five relational database tables. This mapping then exposes the power and expressivity of SQL for querying. Additional flexibility is added by abstracting some search operations, particularly full-text-searching, using R functions.

## Implementation

A parser was developed in Hypertext Preprocessor (PHP, http://www.php.net) according the XML schemas of the objects to extract essential object elements and attributes from each XML submission file. All data in SRAmetadb are faithfully parsed from SRA submissions and no attempt is made to curate, semantically recode, or otherwise clean up SRA data. All field names are also taken from SRA records except for minor changes to improve usability in SQL queries. Fields containing multiple records are generally stored as delimited text within the same record; this denormalization significantly reduces complexity and improves efficiency of queries. The RSQLite package [5] includes an embedded SQLite database engine with Full Text Search (fts3) enabled and can interact with any SQLite database and perform full text search. The SQLite version of SRAmetadb is maintained and distributed for local installation and can be used from R as described below in the examples or independently from other clients (python, perl, java, etc.).

## Results and discussion

In the following sections, we will give an overview of the functionality of the SRAdb package starting with installation, then querying of SRA metadata, retrieval of SRA data based on query results, and finally an example of how to control the IGV browser from within R.

### Installation

Installation of SRAdb can be performed from the R graphical user interface or, more commonly, from the R command line. source (" http://bioconductor.org/biocLite.R")biocLite("SRAdb")

### Example usage

After installing the SRAdb package into R, we load the SRAdb library. library(SRAdb)

The SRAdb R package is a set of functions meant to interact with a SQLite database, so it is necessary to download the SQLite database file before proceeding. This download process can take quite some time on a slow network connection, but the download itself needs to be done only once (or periodically to get updated SRA information). The following code downloads the most recent SRAdb database file and then connects to it from R. sqlfile = getSRAdbFile()sra_con = dbConnect(SQLite(), sqlfile)

#### Full text search

Before accessing SRA data, one must find the appropriate records. SRAdb supports both regular table and field specific SQL commands and a more simplified full-text-searching function. SQLite has a very handy full text search module, fts3, which allow users to do Google-like search with terms and operators. The function getSRA does full text search against all fields in a fts3 table with terms constructed with the Standard Query Syntax and Enhanced Query Syntax (See http://www.sqlite.org/fts3.html for detail). As a simple example, we aim to find all run and study combined records in which any given fields have “breast” and “cancer” words: rs = getSRA(search_terms = "breast cancer", out_types = c("run", "study"), sra_con = sra_con)

Find study records containing a word beginning with “Carcino”: rs <- getSRA(search_terms = "Carcino⋆", out_types = c("study"), sra_con = sra_con)

It is also possible to be more specific to find records containing exact phrase of “breast cancer”, in which “breast” and “cancer” much occur together. In each case, the variable rs will be an R data.frame that can be further processed or filtered in R. The columns in the data.frame are dictated by the “out_types” argument. Representative columns of the resulting data.frame are shown in the following code example. rs <- getSRA(search_terms = "∖"breast cancer∖"", out_types = c("run", "study"),sra_con = sra_con)head(rs[, c(1:5, 7)])## run_alias run run_date updated_date spots run_center## 1 HMEC_2 DRR000109 2008-12-22 2012-06-23 19300723 KUGSPS## 2 MDAMB231_2 DRR000103 2008-12-22 2012-06-23 6447854 KUGSPS## 3 MCF7_3 DRR000110 2008-12-22 2012-06-23 5396205 KUGSPS## 4 T47D_I DRR000106 2008-12-22 2012-06-23 6272361 KUGSPS## 5 MCF7_1_paired DRR000093 2008-12-22 2012-09-06 6141309 KUGSPS## 6 MDAMB231_1 DRR000095 2008-12-22 2012-09-06 6110876 KUGSPS

#### Arbitrary SQL queries

The getSRA function hides most of the detais of the underlying SQLite database and queries. However, one of the strengths of SRAdb is in exposing the SRA metadata via a standard query language, SQL. The column descriptions for the SRAdb SQLite database schema are given in table form in Additional file 1 and are also available via the colDescriptions function in the SRAdb package. As a simple example, we can query SRAdb for the number of runs for each available library strategy (WGS, exome sequencing, RNA-seq, etc.). rs <- dbGetQuery(sra_con, paste("SELECT library_strategy AS 'Library Strategy',","count( ⋆ ) AS Runs FROM ‘experiment‘", "GROUP BY library_strategy order by Runs DESC",sep = " "))head(rs)## Library Strategy Runs## 1 <NA> 90831## 2 WXS 42381## 3 WGS 41033## 4 OTHER 19021## 5 RNA-Seq 9622## 6 AMPLICON 9298

Arbitrarily complex SQL queries can be performed with results returned as an R data.frame. Further filtering, processing, ane even visualization (plotting results) can then be performed using standard R functions.

#### Retrieving data file information

Searching SRA metadata using the SRAdb full-text-search or arbitrary SQL queries results in one or more SRA accessions. Given these accessions, the SRAdb package can determine the associated fastq files for download; in some cases, there may be dozens of fastq files for an accession. As an example, we list fastq file names including ftp addresses associated with “SRX000122”: rs <- listSRAfile("SRX000122", sra_con = sra_con, fileType = "fastq", srcType = "ftp")nrow(rs)## [1] 6

The listSRAfile function does not check file availability, size and date of the fastq files on the server (to avoid multiple ftp roundtrips), but the function getFASTQinfo does this, which is good to know if preparing to download a large file set. rs <- getFASTQinfo(in_acc = c("SRX000122"), srcType = "ftp")

The above commands get fastq file information and essential associated meta data from EBI ENA web site. The following commands will retrieve ‘lite.sra’ file size information from NCBI SRA ftp site: rs <- getSRAinfo(in_acc = c("SRS012041", "SRS000290"), sra_con = sra_con, sraType = "litesra")

The getFASTQfile and getSRAfile functions actually download all available fastq or sra files associated with a set of SRA accessions. For example, to download all fastq files for “SRR000648” and “SRR000657” from EBI ENA site ftp site to a new folder in current directory: getFASTQfile(in_acc = c("SRR000648", "SRR000657"), destDir = getwd(), srcType = "ftp")

Currently both NCBI and EBI support the high-speed fasp protocol for downloading SRA data files and SRAdb has included the functionality for using the protocol. Advanced users are encouraged to use the fasp protocol, but it requires third-party software installation prior to use.

### Interface to IGV

Once raw sequence data are processed by external programs (such as alignment algorithms) or using Bioconductor tools in R, there is often a need to visualize the results in genomic context. The IGV browser serves this purpose nicely. IGV offers an API that allows bidirectional communication between IGV and third-party software like R. The SRAdb package has R functions, summarized in Table 1 that can be used to control IGV. exampleBams = file.path(system.file ("extdata", package = "SRAdb"), dir(system.file("extdata",package = "SRAdb"), pattern = "bam$"))sock <- IGVsocket()IGVgenome(sock, "hg18")IGVload(sock, exampleBams)IGVgoto(sock, "chr1:1-1000")

**Table 1:** Functions to interact with the Integrated Genome Viewer (IGV)

**Function**	**Description**
startIGV	Start IGV from R with different
	amount maximum memory support
IGVsession	Create an IGV session file
IGVgenome	Set the IGV genome
IGVload	Load data into IGV via remote port call
IGVclear	Clear IGV tracks loaded
IGVcollapse	Collapse tracks in the IGV
IGVgoto	Go to a specified region in IGV
IGVsnapshot	Make a file snapshot of the current IGV screen
IGVsocket	Create a Socket Connection to IGV
IGVsort	Sort an alignment track by the specified option

In the short IGV code example, which requires a running IGV session, the IGV genome is first set to “hg1” and example BAM files are then loaded. IGVgoto moves the browser position to chr1:1-1000. Finally, a screen shot is captured to disk for later review. Other combinations of R and IGV commands allow interactive visualization of data driven by R results.

## Conclusions

The public SRA database is the de facto standard location for depositing public sequence data. Bioconductor and R comprise a set of tools for the comprehension of genomic data, including sequence data. Therefore, it is natural to want to be able to quickly gather sequence datasets of interest, download data, and then visualize the results of an analysis. The SRAdb package provides this functionality for the R and Bioconductor community. Furthermore, the SQLite database file can be used by any software with the ability to connect to a SQLite database making for a resource of even broader interest.

## Availability and requirements

**Operating systems**: Windows, Mac OS, Linux, and any other operating systems supporting R.**Programming language**: R**License**: Artistic-2.0**Restrictions to use by non-academics**: None**Source code**: The SRAdb package is available via the Bioconductor [3] website at http://bioconductor.org. Current release version: http://www.bioconductor.org/packages/release/bioc/html/SRAdb.html. The SQLite database itself is available for independent download at http://gbnci.abcc.ncifcrf.gov/backup/SRAmetadb.sqlite.gz.

## Competing interests

The authors declare that they have no competing interests.

## Authors’ contributions

YZ and SD wrote code and documentation and drafted the manuscript. PM and RS supervised the work. All authors read and approved the final manuscript.

## Supplementary Material

Additional file 1 SQLite column descriptions. Column descriptions from the SRAdb SQLite file can be generated with the colDescriptions function in the SRAdb package. Such a reference is quite useful when working in SQL directly. Click here for file
