# Supplementary material for: SRAdb: query and use public next-generation sequencing data from within R
Source: BMC Bioinformatics. 2013 Jan 17;14:19. doi: 10.1186/1471-2105-14-19 (PMC3560148; doi:10.1186/1471-2105-14-19)
Supplement: Additional file 1 — SQLite column descriptions. Column descriptions from the SRAdb SQLite file can be generated with the colDescriptions function in the SRAdb package. Such a reference is quite useful when working in SQL directly. [file 1471-2105-14-19-S1.pdf]

Database Table	Column	Description
submission	ID	
submission	accession	The document's accession as assigned by the Home Archive.
submission	alias	Sbmitter supplied name or id for the submission. This value can be used by the submitterto track a submission without waiting for the Archive to assign an accession.
submission	submission_comment	Submitter assigned comment.
submission	files	Files included in the submission.
submission	center_name	Center name of submitter.
submission	broker_name	Broker authority of this document. If not provided, then the broker is considered "direct".
submission	sra_link	
submission	xref_link	
submission	entrez_link	
submission	ddbj_link	
submission	ena_link	
submission	lab_name	Laboratory name within submitting institution.
submission	submission_date	Submitter assigned preparation date of this submission object.
submission	date_updated	Datetime the record synchronized with SRA, ERA and DRA
study	ID	
study	alias	Submitter designated name of the SRA document of this type. At minimum alias should be unique throughout the submission of this document type. If center_name is specified, the name should be unique in all submissions from that center of this document type.
study	accession	The document's accession as assigned by the Home Archive.
study	study_title	Title of the study as would be used in a publication.
study	study_type	The STUDY_TYPE presents a controlled vocabulary for expressing the overall purpose of the study.
study	study_abstract	Briefly describes the goals, purpose, and scope of the Study. This need not be listed if it can be inherited from a referenced publication.
study	center_project_name	Submitter defined project name. This field is intended for backward tracking of the study record to the submitter's LIMS.
study	study_description	More extensive free-form description of the study.
study	related_studies	Related study or project record from a list of supported databases. The study's information is derived from this project record rather than stored as first class information.
study	primary_study	Whether this study object is designated as the primary source of the study or project information.
study	broker_name	Broker authority of this document. If not provided, then the broker is considered "direct".
study	sra_link	
study	xref_link	
study	entrez_link	
study	ddbj_link	
study	ena_link	
study	url_link	Text label to display for the link.
study	entrez_link	NCBI controlled vocabulary of permitted cross references. Please see http://www.ncbi.nlm.nih.gov/entrez/eutils/einfo.fcgi? .
study	study_attribute	Properties and attributes of the study. These can be entered as free-form tag-value pairs. For certain studies, submitters may be asked to follow a community established ontology when describing the work.
study	submission_accession	Submission accession
study	date_updated	Datetime the record synchronized with SRA, ERA and DRA
sample	ID	
sample	alias	Submitter designated name of the SRA document of this type. At minimum alias should be unique throughout the submission of this document type. If center_name is specified, the name should be unique in all submissions from that center of this document type.
sample	accession	The document's accession as assigned by the Home Archive.

sample	taxon_id	NCBI Taxonomy Identifier. This is appropriate for individual organisms and some environmental samples.
sample	common_name	GenBank common name of the organism. Examples: human, mouse.
sample	anonymized_name	Anonymous public name of the sample. For example, HapMap human isolate NA12878.
sample	scientific_name	Scientific name of sample that distinguishes its taxonomy. Please use a name or synonym that is tracked in the INSDC Taxonomy database. Also, this field can be used to confirm the TAXON_ID setting.
sample	individual_name	Individual name of the sample. This field can be used to identify the individual identity of a sample where appropriate (this is usually NOT appropriate for human subjects). Example: ”Glennie” the platypus.
sample	description	Free-form text describing the sample, its origin, and its method of isolation.
sample	url_link	Text label to display for the link.
sample	entrez_link	NCBI controlled vocabulary of permitted cross references. Please see http://www.ncbi.nlm.nih.gov/entrez/eutils/einfo.fcgi? .
sample	sample_attribute	Properties and attributes of a sample. These can be entered as free-form tag-value pairs. For certain studies, submitters may be asked to follow a community established ontology when describing the work.
sample	submission_accession	Submission accession
sample	broker_name	Broker authority of this document. If not provided, then the broker is considered ”direct”.
sample	sra_link	
sample	xref_link	
sample	entrez_link	
sample	ddbj_link	
sample	ena_link	
sample	date_updated	Datetime the record synchronized with SRA, ERA and DRA
experiment	ID	
experiment	bamFile	Available converted bam file name
experiment	fastqFTP	ftp to fastq file
experiment	alias	Submitter designated name of the SRA document of this type. At minimum alias should be unique throughout the submission of this document type. If center_name is specified, the name should be unique in all submissions from that center of this document type.
experiment	accession	The document’s accession as assigned by the Home Archive.
experiment	center_name	Owner authority of this document and namespace for submitter’s name of this document. If not provided, then the submitter is regarded as ’Individual’ and document resolution can only happen within the submission.
experiment	title	Short text that can be used to call out experiment records in searches or in displays. This element is technically optional but should be used for all new records.
experiment	study_name	Study name.
experiment	study_accession	Study accession.
experiment	design_description	More details about the setup and goals of the experiment as supplied by the Investigator.
experiment	sample_name	Sample name.
experiment	sample_accession	Sample accession.
experiment	sample_member	
experiment	library_name	The submitter’s name for this library.
experiment	library_strategy	Sequencing technique intended for this library.
experiment	library_source	The LIBRARY_SOURCE specifies the type of source material that is being sequenced.
experiment	library_selection	Whether any method was used to select and/or enrich the material being sequenced.
experiment	library_layout	LIBRARY_LAYOUT specifies whether to expect single, paired, or other configuration of reads. In the case of paired reads, information about the relative distance and orientation is specified.

experiment	targeted_loci	
experiment	library_construction_protocol	Free form text describing the protocol by which the sequencing library was constructed.
experiment	spot_length	
experiment	adapter_spec	Some technologies will require knowledge of the sequencing adapter or the last base of the adapter in order to decode the spot.
experiment	read_spec	Contains READ_INDEX, READ_LABEL, READ_CLASS, READ_TYPE, BASE_COORD, RELATIVE_ORDER, CYCLE_COORD, EXPECTED_BASECALL, EXPECTED_BASECALL_TABLE, BASECALL
experiment	platform	The PLATFORM record selects which sequencing platform and platform-specific runtime parameters. This will be determined by the Center.
experiment	instrument_model	Instrument model for a platform.
experiment	platform_parameters	
experiment	sequence_space	Base Space or Color Space
experiment	base_caller	Name and version of the base or color calling software.
experiment	quality_scorer	Name and version of the quality scoring software.
experiment	number_of_levels	DEPRECATED. Number of distinct values possible with this scoring system.
experiment	multiplier	DEPRECATED.
experiment	qtype	phred or other
experiment	url_link	The internet service link (file:, http:, ftp:, etc).
experiment	entrez_link	NCBI controlled vocabulary of permitted cross references. Please see http://www.ncbi.nlm.nih.gov/entrez/eutils/einfo.fcgi? .
experiment	experiment_attribute	Properties and attributes of the experiment. These can be entered as free-form tag-value pairs.
experiment	submission_accession	Submission accession
experiment	broker_name	Broker authority of this document. If not provided, then the broker is considered ”direct”.
experiment	sra_link	
experiment	xref_link	
experiment	entrez_link	
experiment	ddbj_link	
experiment	ena_link	
experiment	date_updated	Datetime the record synchronized with SRA, ERA and DRA
run	ID	
run	bamFile	Available converted bam file name
run	fastqFTP	ftp to fastq file
run	alias	Submitter designated name of the SRA document of this type. At minimum alias should be unique throughout the submission of this document type. If center_name is specified, the name should be unique in all submissions from that center of this document type.
run	accession	The document's accession as assigned by the Home Archive.
run	instrument_name	Center-assigned name or id of the instrument used in the run.
run	run_date	ISO date when the run took place.
run	run_file	DEPRECATED. Name of the submission file containing the run. This file may have included multiple runs.
run	run_center	If applicable, the name of the contract sequencing center that executed the run. Example: 454MSC.
run	total_data_blocks	DEPRECATED. The number of data blocks to expect in this run.
run	experiment_accession	Experiment accession
run	experiment_name	Experiment name
run	url_link	The internet service link (file:, http:, ftp:, etc).
run	entrez_link	NCBI controlled vocabulary of permitted cross references. Please see http://www.ncbi.nlm.nih.gov/entrez/eutils/einfo.fcgi? .
run	run_attribute	Properties and attributes of a RUN. These can be entered as free-form tag-value pairs. For certain studies, submitters may be asked to follow a community established ontology when describing the work.

run	submission_accession	Submission accession
run	broker_name	Broker authority of this document. If not provided, then the broker is considered "direct".
run	sra_link	
run	xref_link	
run	entrez_link	
run	ddbj_link	
run	ena_link	
run	date_updated	Datetime the record synchronized with SRA, ERA and DRA
data_block	ID	
data_block	name	The plate/slide/flowcell name for this data block. (454) plate name (Illumina) flowcell name (SOLiD) slide name (Helicos) flowcell
data_block	sector	Higher level partition of run data to which this data block pertains. (454) not used (Illumina) Lane number (SOLiD) slide (Helicos) channel
data_block	region	Lower level partition of run data to which this data block pertains, typically the field of view for the imaging camera. (454) 0 if whole plate is used, 1..16 for gasket partition. (Illumina) Tile number (1..200+), or use 0 if file contains all tiles. (SOLiD) Panel number (1..4096), or use 0 if file contains all panels.
data_block	files	Actual run data are contained in one of the files listed in the submission manifest. Each data block is represented by one SRF file, one SFF file, one compressed fastq file, or one compressed tar archive file.
data_block	run_accession	Submission accession
data_block	date_updated	Datetime the record synchronized with SRA, ERA and DRA
